# Bendici’s Test for Sugar

**Published:** 1913-02

**Authors:** 


BENDICI'S TEST FOR SUGAR.—Myers, *Munch. Med. Woch.*, endorses this test as more delicate to sugar and less to other reducing substances in urine, than Fehling's, Haine's and analogens copper solutions.

| | | |
|---|---|---|
| Sodium or Potassium citrate | 173 | grammes |
| Sodium carbonate, crystallized | 200 | " |
| (or anhydrous 100 grammes) | | |
| Boiling water | 800 | " |

Cool and filter, then add
Cupric sulphate .................. 17.3 grammes
Water ..........................100. "
Dilute to one Liter. Use exactly as Fehling's solution.

---

Perforation by Proctoscope.—O. Retzloff, *Münch. Med. Woch.,* July 23, 1912, reports a case which recovered after suture.

